# Whey Protein-Based Micro- and Nanocapsules Loaded with Quercetin: Design and Functional Properties

**DOI:** 10.3390/ijms27156744

**Published:** 2026-07-28

**Authors:** Adrian Haranguș, Irina Camelia Chiș, Simona Valeria Clichici, Șoimița Suciu, Sonia Balint, Gertrud Alexandra Paltinean, Ioan Petean, Doriana Maria Popa, Teodora Mocan

**Affiliations:** 1 Division of Physiology, Department of Morpho-Functional Sciences, “Iuliu Hatieganu” University of Medicine and Pharmacy, 1-3 Clinicilor Street, 400006 Cluj-Napoca, Romania; harangus_adrian@elearn.umfcluj.ro (A.H.); sclichici@umfcluj.ro (S.V.C.); mihaela.suciu@umfcluj.ro (Ș.S.); teodoramocan@umfcluj.ro (T.M.); 2 Raluca Ripan Institute for Research in Chemistry, Babes-Bolyai University, 30 Fantanele Street, 400294 Cluj-Napoca, Romania; sonia.balint@ubbcluj.ro; 3 Faculty of Chemistry and Chemical Engineering, Babes-Bolyai University, 11 Arany Janos Street, 400028 Cluj-Napoca, Romania; ioan.petean@ubbcluj.ro; 4 Alba Iulia County Emergency Hospital, 510077 Alba Iulia, Romania; popa.dorianaa@yahoo.com; 5 General Nursing, Department of Pediatrics and Specialized Pediatric Nursing, Faculty of Law and Social Sciences, “1 Decembrie 1918” University of Alba Iulia, 5 Gabriel Bethlen Street, 510009 Alba Iulia, Romania; 6 Department of Nanomedicine, Regional Institute of Gastroenterology and Hepatology, 5 Constanta Street, 400158 Cluj-Napoca, Romania

**Keywords:** microspheres, nanocapsules, whey–quercetin, S/O/W methods, interfacial polymerization methods

## Abstract

Whey–quercetin-loaded microspheres were synthesized using the solid/organic phase/water phase (S/O/W) method, whereas nanocapsules were prepared by interfacial polymerization. The resulting systems noted that Q1, Q2, and Q3 were characterized by polarized light microscopy (PLM), scanning electron microscopy (SEM), atomic force microscopy (AFM), Fourier transform infrared (FTIR) spectroscopy, X-ray diffraction (XRD), differential scanning calorimetry (DSC), high-performance liquid chromatography (HPLC), and antioxidant activity assays. Structural and spectroscopic analyses confirmed the successful encapsulation of whey and quercetin within both microsphere and nanocapsule systems. Q2 exhibited the highest encapsulation efficiency (51.79 ± 1.03%) and loading capacity (5.78 ± 0.71%), demonstrating its superior quercetin encapsulation performance. However, measurable antioxidant activity was detected exclusively in the nanocapsule formulations, whereas microspheres showed no detectable activity under the experimental conditions. Although the antioxidant activity of encapsulated quercetin was lower than that of free quercetin, the nanocapsules retained a radical-scavenging capacity, supporting their potential as effective delivery systems.

## 1. Introduction

**Figure 1:**
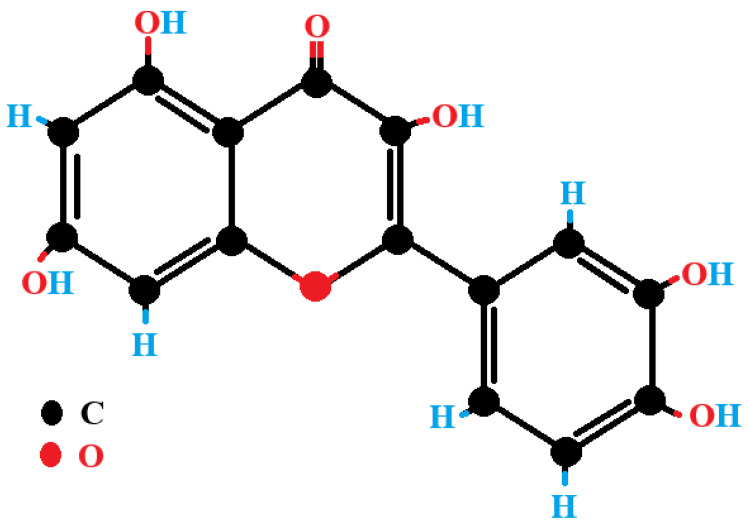
Chemical formula of quercetin.

Quercetin is a naturally occurring flavonol widely distributed in fruits and vegetables and is recognized as one of the most biologically active dietary polyphenols [1,2,3,4]. As illustrated in Figure 1, its structure consists of two aromatic rings linked by a heterocyclic ring and contains five hydroxyl groups that largely determine its biological activity through hydrogen bonding, free radical scavenging, and metal ion chelation [5,6].

Owing to these structural characteristics, quercetin exhibits remarkable antioxidant properties and has attracted considerable attention for its potential use in nutraceutical, pharmaceutical, and functional food applications [7,8,9,10,11].

The antioxidant activity of quercetin has been extensively demonstrated using various in vitro assays, including DPPH, ABTS, hydroxyl radical, nitric oxide, hydrogen peroxide, and superoxide radical scavenging systems [12,13,14,15]. Through its ability to neutralize reactive oxygen and nitrogen species, quercetin contributes to the reduction in oxidative stress, which is involved in the development of numerous chronic disorders [16]. Consequently, quercetin has been associated with anti-inflammatory, anticancer, hepatoprotective, cardioprotective, neuroprotective, nephroprotective, and antidiabetic effects in experimental studies [8,9,10,11,12,13,14,15,16,17,18,19,20,21,22,23,24,25,26]. Despite these promising biological properties, its practical application remains limited by poor aqueous solubility, low chemical stability, rapid degradation under physiological conditions, and limited oral bioavailability [27,28,29].

To overcome these limitations, considerable efforts have been devoted to the development of encapsulation systems capable of protecting quercetin from degradation while improving its solubility, stability, bioaccessibility, and controlled release [30,31,32,33]. Micro- and nanoencapsulation have emerged as effective strategies for enhancing the physicochemical properties of poorly soluble bioactive compounds, allowing for improved protection against environmental factors while enabling controlled or targeted delivery depending on the carrier design [32,33,34,35].

Among the different encapsulating materials, protein- and polysaccharide-based carriers have attracted particular interest because of their biocompatibility, biodegradability, and suitability for food and pharmaceutical applications [36,37]. Protein-based systems, including whey proteins, casein, zein, and soy proteins, can efficiently bind polyphenols through hydrophobic interactions and hydrogen bonding, leading to improved encapsulation efficiency, enhanced aqueous dispersibility, and the increased bioaccessibility of flavonoids [30,32,33,34,35]. In parallel, polysaccharide-based carriers such as chitosan, alginate, pectin, and gum Arabic provide excellent mucoadhesive properties, protection during gastrointestinal transit, and sustained-release behavior [38,39,40]. More recently, hybrid protein–polysaccharide delivery systems have been developed to combine the advantages of both materials, resulting in improved physicochemical stability, encapsulation efficiency, and the controlled release of bioactive compounds [34].

Among the protein-based carriers, whey protein isolate (WPI) has emerged as one of the most promising encapsulating materials because of its excellent emulsifying, gel-forming and film-forming properties, together with its high nutritional value, biocompatibility, and biodegradability [32,33,34,35]. Whey proteins readily interact with quercetin through non-covalent interactions, promoting the formation of stable micro- and nanostructures capable of protecting the flavonoid against oxidation and improving its physicochemical stability [30,31]. Previous studies have demonstrated that whey protein-based delivery systems enhance quercetin stability during processing and simulated gastrointestinal digestion while increasing its bioaccessibility, highlighting their potential for nutraceutical and functional food applications [41,42].

Despite the growing number of studies on quercetin encapsulation, direct comparisons between micro- and nanoscale whey-based delivery systems prepared using different encapsulation approaches remain limited [43,44]. Understanding how the encapsulation strategy influences morphology, structural organization, encapsulation efficiency, and antioxidant performance is essential for optimizing carrier design according to the intended application.

Therefore, the present study aimed to develop whey–quercetin micro- and nanocapsules using two different encapsulation approaches and to comparatively evaluate their physicochemical properties, morphology, structural organization, encapsulation efficiency, and antioxidant activity (outlined in Table 1). The obtained results provide insights into the relationship between formulation characteristics and the performance of whey protein-based delivery systems for quercetin.

## 2. Results

### 2.1. Morphological Characterization of Quercetin and Whey (SEM)

**Figure 2:**
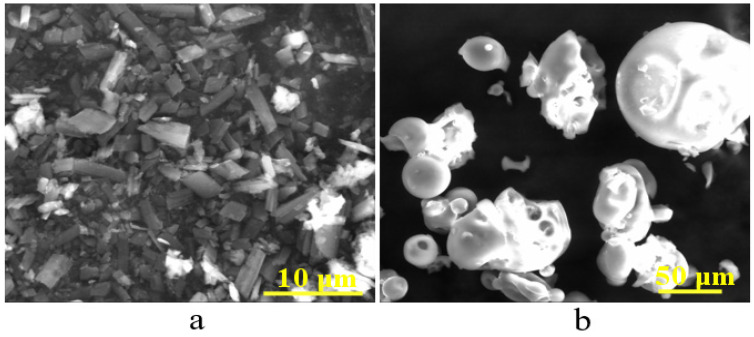
SEM images of (**a**) quercetin and (**b**) whey.

Additional investigations were performed on quercetin and whey individually to evaluate their morphological characteristics, as illustrated in Figure 2. The SEM micro-graph of quercetin (Figure 2a) reveals agglomerates composed of irregular prismatic structures with smooth surfaces, which may be attributed to their high surface energy [31]. The prismatic particles exhibited average lengths ranging from 4 to 6 µm and widths between 1 and 3 µm.

The SEM image of whey (Figure 2b) shows a tendency to form spherical particles with smooth surface topography and the presence of central cavities. This morphology may be attributed to physical processes occurring during processing and drying [45,46,47,48]. The spherical particles exhibited diameters ranging from 15 to 116 µm, while the central cavities ranged from 5 to 10 µm.

### 2.2. FTIR Spectroscopy of Microspheres and Nanocapsules

**Figure 3:**
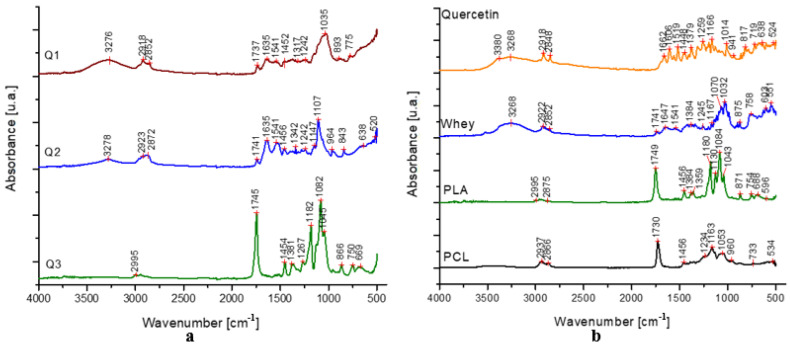
FTIR spectra of (**a**) Q1, Q2 nanocapsules, and Q3 microspheres and (**b**) quercetin, whey and PLA, and PCL polymers.

Fourier transform infrared (FTIR) spectroscopy was employed to identify the functional groups and confirm the formation of PLA microspheres (Q3) loaded with the whey–quercetin complex, as well as nanocapsules prepared by interfacial polymerization (Q1, Q2). The composition and preparation strategy of the Q1, Q2, and Q3 formulations are summarized in Table 2. The corresponding absorption spectra are presented in Figure 3.

The FTIR spectrum of quercetin showed characteristic absorption bands associated with its flavonoid structure. The broad band observed at approximately 3311 cm^−1^ was attributed to O–H stretching vibrations of phenolic hydroxyl groups involved in hydrogen bonding. The bands at 1606 and 1519 cm^−1^ corresponded mainly to conjugated carbonyl/C=C aromatic vibrations and aromatic ring stretching, respectively. Additional bands observed at 1259 and 1318 cm^−1^ were associated with C–O stretching vibrations of phenolic groups, confirming the characteristic structure of quercetin [31,49,50].

The FTIR spectra of Q1 and Q2 exhibited characteristic whey protein absorption bands. The band located around 1635 cm^−1^ was assigned to the amide I region, mainly related to C=O stretching vibrations of peptide bonds, while the band at approximately 1541 cm^−1^ corresponded to the amide II region, associated with N–H bending and C–N stretching vibrations. The slightly higher intensity of these bands in Q2 is consistent with its higher whey protein content [30]. The whey protein spectrum showed typical absorption bands at approximately 3255 cm^−1^ (amide A region), 1647 cm^−1^ (amide I), and 1540 cm^−1^ (amide II), confirming the presence of protein structures within the formulations [51].

For the polymeric components, the characteristic ester carbonyl stretching band of PCL was observed at approximately 1730–1742 cm^−1^. The slight variation in its position may be related to changes in the polymer microenvironment caused by the incorporation of whey and quercetin. In the spectra of Q3 and PLA, the bands observed at approximately 1745–1749 cm^−1^ were attributed to ester C=O stretching vibrations. The bands at 1454–1455 cm^−1^ corresponded to CH_3_ bending vibrations, while the signals at 1182 and 1081 cm^−1^ were assigned to the C–O–C and C–O stretching vibrations of ester groups. These characteristic peaks confirm the presence of the PLA-based polymeric matrix in Q3 [52].

### 2.3. Optical Microscopy and SEM Analysis for Microspheres and Nanocapsules

**Figure 4:**
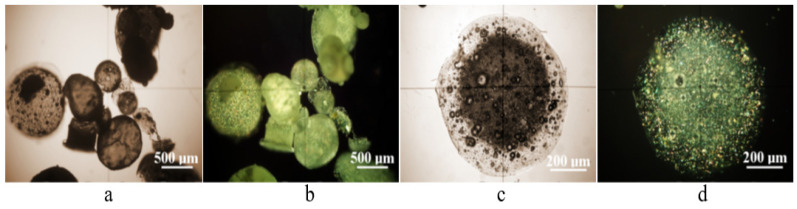
Polarized light microscopy of PLA microspheres loaded with whey and quercetin. (**a**,**b**) Overview in transmitted and polarized light; (**c**,**d**) structural detail in transmitted and polarized light.

Microspheres obtained by the S/O/W method exhibited a wide size distribution (Figure 4), with diameters ranging from 300 to 1000 µm. Transmitted light microscopy, Figure 4a, showed predominantly spherical particles with porous structures, while polarized light, Figure 4b, revealed a light-yellow coloration.

**Figure 5:**
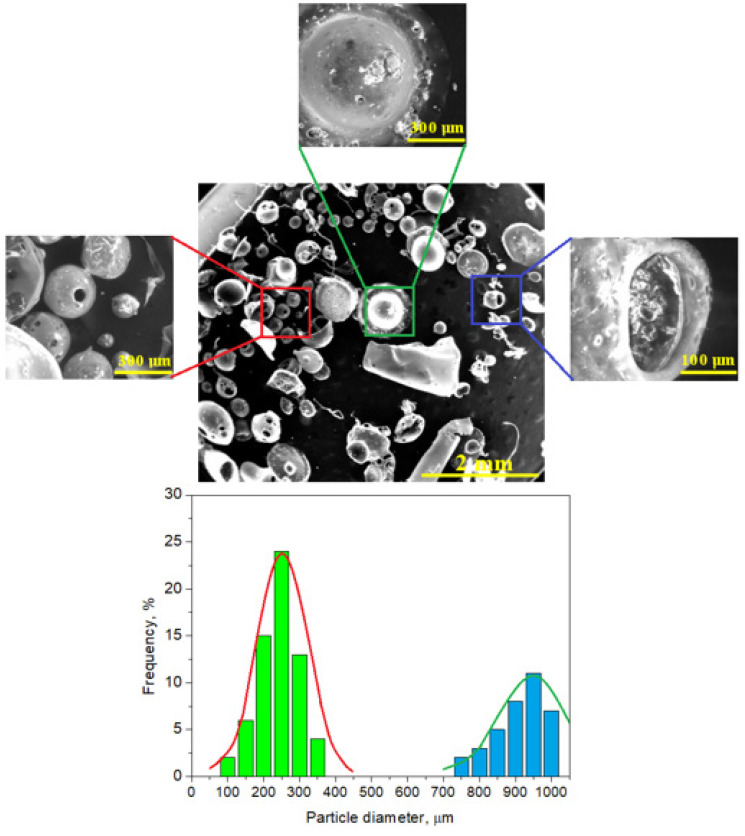
SEM images of PLA microspheres (Q3) loaded with whey and quercetin and the particle size distribution histogram.

The structural features of the microspheres are illustrated in Figure 4c,d. The images revealed a dense surface enriched with whey and quercetin, which was particularly evident under polarized light microscopy, where these components exhibited a distinctive multicolored appearance. The microsphere exhibited an average diameter of approximately 500 µm. Detailed SEM analysis (Figure 5) revealed a heterogeneous population of microspheres with both porous and smooth surface morphologies. Their diameters ranged from 90 to 950 µm. In the red-marked region, the pore diameters varied between 30 and 90 µm, whereas the microsphere diameters ranged from 90 to 300 µm.

The dimensional heterogeneity of the microsphere powder is evident from the SEM images presented in Figure 5, which provide detailed information on their microstructure. Particle size analysis was performed using ImageJ software 1.53K, and the resulting particle size distribution is shown in Figure 5. The distribution exhibited a bimodal pattern with two predominant particle populations. The finer fraction was centered around 250 µm, whereas the coarser fraction was centered around 950 µm. The smallest structural features located at the periphery of the microspheres were not included in the particle size analysis. However, higher-magnification SEM images revealed secondary spherical structures with diameters of approximately 10–12 µm. These features were not detected because they were below the minimum particle size threshold defined for the ImageJ analysis (0–1000 µm, using a particle-size class interval of 50 µm).

**Figure 6:**
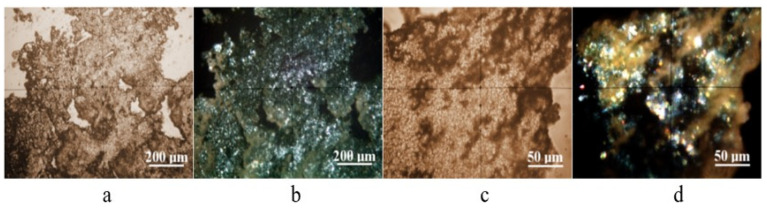
Optical microscopy of whey–quercetin nanocapsules: Panels (Q1 (**a**,**b**)) show overviews observed in transmitted light and cross-polarized. Panels (Q2 (**c**,**d**)) present structural details under transmitted light and polarized light.

Nanocapsules obtained by interfacial polymerization are presented in Figure 6. Op-tical microscopy revealed particle distribution and birefringent characteristics; however, the dimensions of individual nanocapsules could not be precisely determined due to their small size.

**Figure 7:**
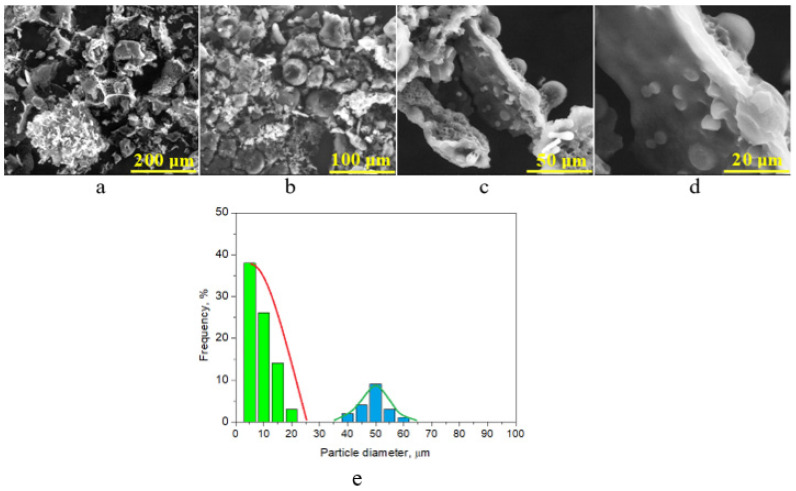
SEM images of whey–quercetin nanocapsules: (**a**,**b**) surface topography of Q2; (**c**,**d**) morphological detailing of Q1; and (**e**) particle size distribution histogram.

SEM images (Figure 7) show the morphology of the nanocapsule samples. Porous domains (5–10 µm) (Figure 7a) were observed. Agglomerated nanocapsules embedded in the matrix, as well as isolated structures, were observed within clusters ranging in diameters from 5 to 55 µm. These dimensions correspond to aggregates of nanocapsules rather than individual particles, indicating a concentrated structural organization.

Higher magnification images (Figure 7c) revealed well-defined nanocapsule domains together with needle-like quercetin crystals measuring approximately 16–18 µm in length. These nanocapsules were more clearly distinguished at higher magnification, as shown in Figure 7d. Particle size analysis performed using ImageJ with a threshold ranging from 0 to 100 µm and a size class of 5 µm (Figure 7e) revealed a bimodal distribution of nanocapsule cluster sizes. The sample was dominated by the finest fractions centered at approximatively 5 µm and below, which accounted for more than 30% of the total composition. This size class corresponds to well-individualized nanocapsule clusters formed through weak interparticle attractive forces and is expected to readily disperse into individual nanocapsules under suitable dispersion conditions, as further confirmed by the AFM analysis. The second population consisted of larger clusters, approximately 50 µm in diameter, comprising nanocapsules that remained attached to the parent polymer matrix together with randomly aggregated nanocapsule clusters. This fraction represented less than 19% of the total particle population. The relatively low proportion of these coarse aggregates indicates good potential for the subsequent dispersion of the nanocapsules.

### 2.4. AFM Analysis

**Figure 8:**
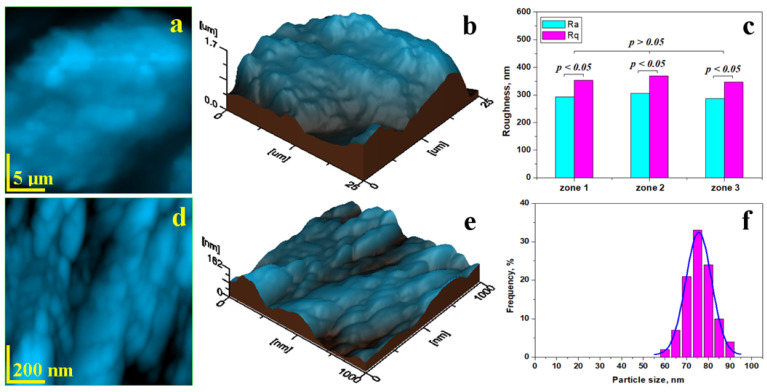
AFM images of whey–quercetin loaded PLA microspheres (Q3): (**a**) topographic image, (**b**) three-dimensional profile, (**c**) surface roughness map at the microstructural level, (**d**) topographic image, (**e**) three-dimensional surface profile, and (**f**) nanoparticle distribution at the nanostructural level.

**Figure 9:**
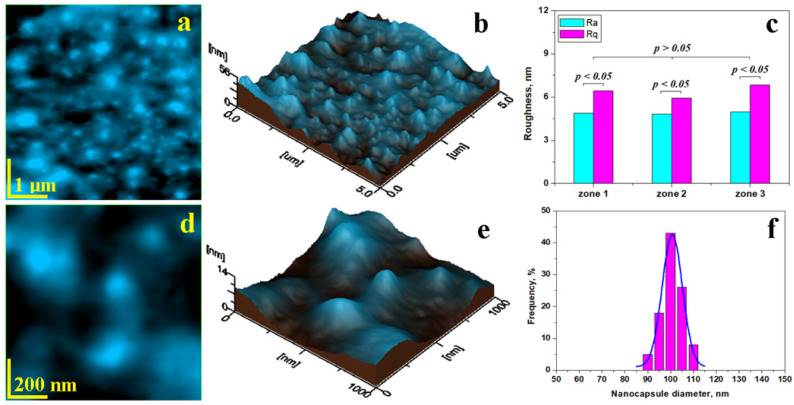
AFM images of the self-assembled thin film of whey–quercetin nanocapsules (Q1): (**a**) topographic image, (**b**) three-dimensional surface profile, and (**c**) surface roughness map at the microstructural level, (**d**) topographic image, (**e**) three-dimensional surface profile, and (**f**) nanocapsule diameter distribution at the nanostructural level.

AFM investigation provided high-resolution topographic details of the microspheres and nanocapsules (Figure 8 and Figure 9).

Microspheres exhibited slightly irregular surfaces. Nanostructural features in the form of alveoli (~75 ± 15 nm) were uniformly distributed on the surface.

Nanocapsules formed uniformly distributed thin films with significantly lower roughness. They exhibited a mean diameter of 100 ± 10 nm and interparticle spacing of 150–200 nm.

### 2.5. Antioxidant Activity

The antioxidant activity was evaluated using the ABTS radical scavenging assay (Table 1). The ABTS•^+^ radical was generated by chemical oxidation with potassium persulfate and stabilized by incubation in the dark. The decrease in absorbance at 415 nm was used to monitor radical scavenging activity.

The antioxidant activity was expressed as IC_50_ values (µg/mL), with lower IC_50_ values indicating higher radical scavenging activity. Quercetin showed the highest antioxidant activity (IC_50_ ≈ 0.5–0.6 µg/mL). The Q1 nanocapsules exhibited moderate activity (IC_50_ = 5.1 µg/mL), whereas Q2 showed lower activity (IC_50_ = 17.6 µg/mL). In contrast, whey and sample Q3 showed no detectable antioxidant activity under the experimental conditions.

**Figure 10:**
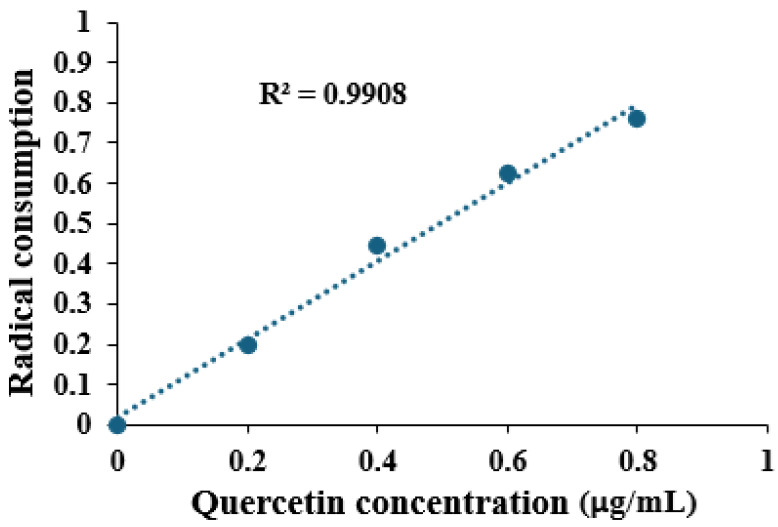
Calibration curve of quercetin for the ABTS•^+^ radical scavenging.

The calibration curve (Figure 10) obtained from quercetin standards showed a linear correlation between concentration and absorbance, allowing for the estimation of quercetin content in the analyzed samples.

The calibration curve (Figure 10) demonstrated good linearity over the concentration range of 0.2–0.8 µg/mL.

**Figure 11:**
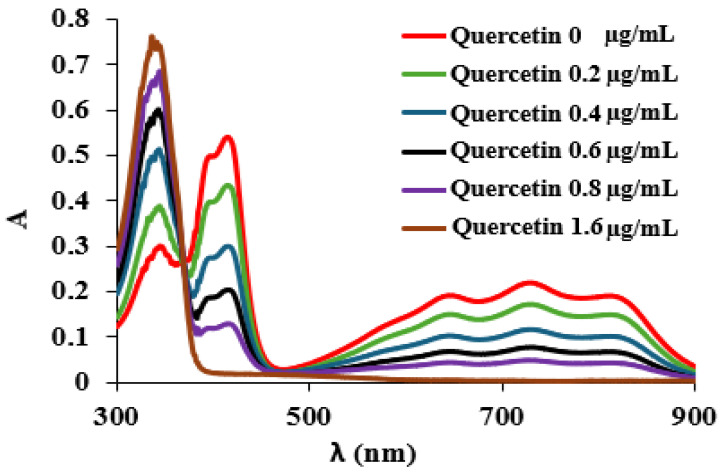
UV–Vis molecular absorption spectra of the ABTS radical in the presence of different quercetin concentrations measured after 30 min of incubation.

Prior to the evaluation of antioxidant activity, UV–Vis analysis (Figure 11) was performed to confirm the presence of quercetin within the investigated formulations and to establish the relationship between absorbance intensity and quercetin concentration. 

UV–Vis spectra (Figure 11) indicated a progressive decrease in absorbance in the 370–900 nm range with increasing quercetin concentration, indicating efficient reduction of the ABTS•^+^ radical. The characteristic absorption band observed at approximately 430 nm confirmed the presence of quercetin in both free and encapsulated forms. This color change from green-blue to colorless further confirmed the antioxidant activity of quercetin.

The results indicate that free quercetin exhibited the strongest ABTS radical scavenging activity, whereas the nanocapsule formulations retained moderate activity depending on their composition. Thus, encapsulation did not enhance the intrinsic antioxidant activity of quercetin in the ABTS assay but preserved measurable radical-scavenging activity in the nanocapsule formulations. Among the nanocapsules, Q1 demonstrated the most promising antioxidant performance, suggesting its potential as a delivery system for applications where controlled release and antioxidant protection are required.

### 2.6. X-Ray Diffraction

**Figure 12:**
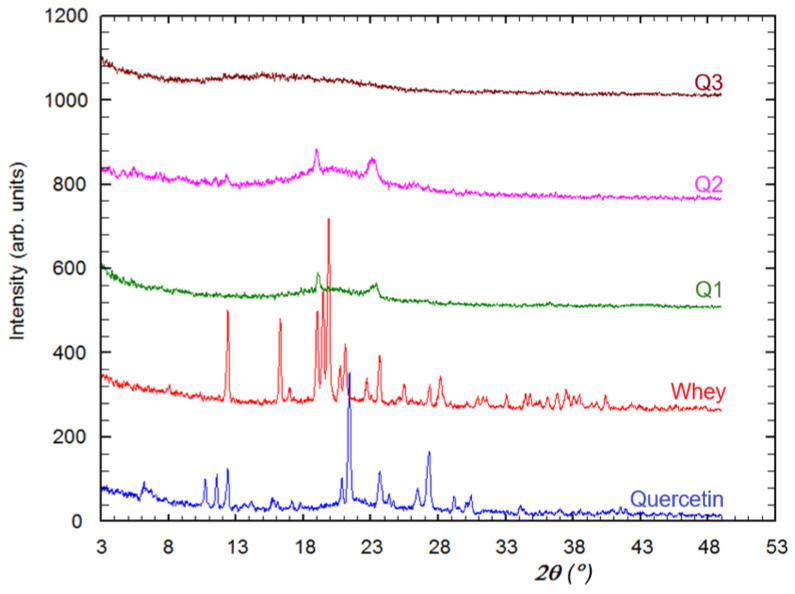
XRD patterns of quercetin, whey, whey–quercetin nanocapsules (Q1 and Q2), and whey–quercetin loaded PLA microspheres (Q3).

The X-ray diffraction (XRD) patterns of quercetin, whey, nanocapsules, and microspheres are presented in Figure 12. The diffraction patterns of quercetin and whey exhibited distinct characteristic peaks, indicating the presence of crystalline domains. The crystalline phase detected in the whey sample was identified as lactose by comparison with the LACTOS01 entry in the Cambridge Structural Database (CSD). In contrast, the XRD patterns of the encapsulated formulations (Q1 and Q2) showed a marked reduction in the intensity of the characteristic quercetin reflections, accompanied by peak broadening and the appearance of a diffuse halo. These changes indicate a decrease in crystallinity and the transformation of quercetin from a crystalline to a predominantly amorphous or molecularly dispersed state within the polymer matrix. The absence of the sharp crystalline peaks of quercetin in the encapsulated samples suggests successful incorporation of the bioactive compound into the polymeric carrier rather than the presence of separate crystalline quercetin particles. Finally, the diffraction pattern of Q3 demonstrates a fully amorphous structure.

### 2.7. HPLC Analysis

HPLC analysis of quercetin content (Table 2) and other polyphenolic compounds was carried out using the method described in [53]. The separation was performed on a Lichrosorb RP-C18 column (25 × 0.46 cm) at a column temperature of 22 °C, with UV detection at 270 nm, and a flow rate of 1 mL/min. The mobile phase was a mixture of methanol (A, HPLC grade), 0.1% formic acid solution (Millipore ultrapure water SAS, Molsheim, France), and a gradient was applied.

For extraction, 2 mg of sample was added to 10 mL of 80% methanol solution. The mixture was stirred and placed in an ultrasonic bath for 60 min to extract the substances of interest. After sonication, the samples were filtered through a 0.45 μm PTFE syringe filter (Chromafil Xtra PTFE, 25 mm, 0.45 μm; Macherey-Nagel, Duren, Germany) and injected into the HPLC system.

The encapsulation efficiency (EE) was calculated using the following equations:

(1) $$\mathrm{EE}\%=\frac{C_{\mathrm{HPLC}\;}\times {\;M}_{\mathrm{nanocapsules}}}{M_{\mathrm{initial}\;\mathrm{Quercetin}}}\;\times 100$$

(2) $$\mathrm{EE}\%=\frac{C_{\mathrm{HPLC}\;}\times {\;M}_{\mathrm{microspheres}}}{M_{\mathrm{initial}\;\mathrm{Quercetin}}}\times 100$$

where

C_HPLC_ is the quercetin content determined with HPLC (mg/g of microspheres and nanocaspules).

M_microspheres_ and M_nanocapsules_ are the total mass of the recovered microspheres/nanocapsules in g.

M_Quercetin_ is the initial amount of quercetin used during formulation.

The total amount of encapsulated quercetin:

(3) $$M_{\mathrm{encapsulated}\;}={\;C}_{\mathrm{HPLC}\;}\times {\;M}_{\mathrm{nanocapsules}}$$

(4) $$M_{\mathrm{encapsulated}\;}={\;C}_{\mathrm{HPLC}\;}\times {\;M}_{\mathrm{microcapsules}}$$

and the loading capacity follows the equation:

(5) $$\mathrm{LC}\%=\frac{M_{\mathrm{encapsulated}\;\mathrm{quercetin}\;}}{M_{\mathrm{nanocapsules}}}\;\times \;100$$

(6) $$\mathrm{LC}\%=\frac{M_{\mathrm{encapsulated}\;\mathrm{quercetin}\;}}{M_{\mathrm{microspheres}}}\times 100$$

where M_encapsulated quercetin_ represents the amount of quercetin incorporated into the microspheres, determined by HPLC analysis (mg), and M_microspheres/nanocapsules_ represents the total mass of dried microspheres (mg).

**Figure 13:**
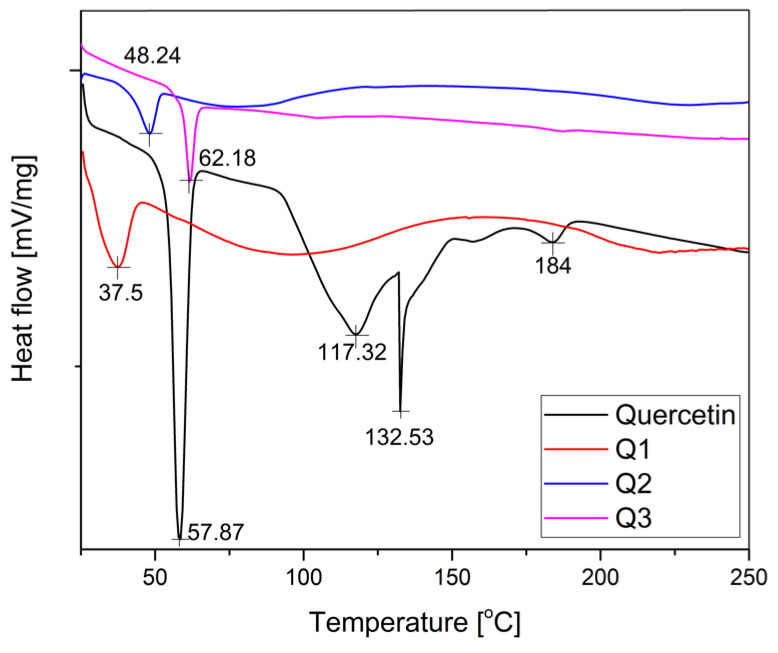
DSC termogram of quercetin, whey–quercetin nanocapsules (Q1 and Q2), and whey–quercetin loaded PLA microspheres (Q3).

The DSC thermogram of pure quercetin exhibited a sharp endothermic melting peak at approximately 57.9 °C (Figure 13), characteristic of its crystalline structure. As can be seen in Table 3, in contrast, formulations Q1 and Q2, prepared by interfacial polymerization, did not exhibit this melting endotherm, suggesting that quercetin was successfully encapsulated and predominantly existed in an amorphous or molecularly dispersed state within the nanocapsules. Similarly, the DSC thermogram of Q3 showed an endothermic peak at approximately 62.2 °C, attributed to the melting of the PLA phase, while the characteristic melting peak of quercetin was absent. These findings indicate that quercetin was effectively incorporated into the PLA microspheres, with a substantial reduction in its crystallinity, reflecting favorable drug–polymer interactions and successful encapsulation [22,42].

## 3. Discussion

The prismatic morphology of quercetin is likely associated with its high surface energy, whereas the spherical morphology of whey particles is attributed to processing and drying conditions [31,48].

FTIR analysis confirmed the successful incorporation of quercetin and whey into the nanocapsules (Q1 and Q2) and PLA microspheres (Q3). The observed shifts in the carbonyl region suggest interactions between the polymer matrix and the encapsulated com-pounds. The higher intensity of characteristic absorption bands in Q2 is consistent with its higher whey content [49,52].

Microscopy analyses demonstrated that the microspheres possessed a heterogeneous and porous structure, which is favorable for encapsulation and controlled release. The presence of internal cavities and multi-scale porosity is expected to facilitate diffusion-based release mechanisms.

In contrast, the nanocapsules exhibited smaller size, better dispersion, and a more uniform morphology, suggesting improved structural organization and enhanced potential as delivery systems.

AFM analysis highlights significant differences in surface roughness between microspheres and nanocapsules. The higher roughness of the microspheres reflects polymer flow and solidification processes, while the smoother surface of the nanocapsules indicates a more homogenous and stable morphology. Owing to their larger diameters, as observed by SEM, the PLA microspheres loaded with whey and quercetin could not be imaged over their entire circumference by AFM. Instead, representative spherical cap regions were analyzed to reveal their surface topography (Figure 8a). The microsphere surface exhibited slight irregularities due to the relative flow of semi-liquid PLA during the encapsulation of the active compounds, which appeared to be uniformly distributed within the composite structure and well-embedded into the PLA matrix. The spherical geometry was more clearly visualized in the three-dimensional topographic image (Figure 8b), which revealed a continuous surface free of visible defects or cracks. Surface roughness measurements were highly reproducible across three independent regions (Figure 8c), with no statistically significant differences among the measurements (*p* > 0.05). As expected, the root mean square roughness (Rq) was consistently higher than the arithmetic average roughness (Ra) owing to the different calculation methods of the two parameters [54]. This difference was statistically significant (*p* < 0.05).

The outermost layer of the microspheres displayed a well-defined nanostructured surface, characterized by fine PLA features formed during the polymer flow throughout the spheroidization process. The whey–quercetin mixture formed nanoscale surface features with an average diameter of 75 ± 15 nm, which were uniformly distributed on the microsphere’s surface (Figure 8d). These nanostructures correspond to the small yellow-green features observed in the topographic image (Figure 8d).

The topographic profile suggests the presence of nanoscale surface depressions that may reflect underlying structural heterogeneity within the outer PLA layer. However, their relationship to the spatial distribution of the encapsulated bioactive compounds cannot be confirmed by AFM alone. The encapsulation strategy involves incorporating the bioactive compound into individual nanocapsules dispersed within the microspheres, an approach that may improve its distribution within the polymer matrix, although this hypothesis requires confirmation through drug-release studies (Figure 9a). The density of nanocapsules within the thin film can be easily controlled through the dispersion concentration; a high concentration will generate a dense thin film while a diluted dispersion allows for increased spacing between nanocapsules. The three-dimensional profile in Figure 9b revealed a uniform distribution without local clustering and a smooth overall appearance. Thus, the surface roughness was significantly lower than on the microsphere assemblies. The nanocapsule thin film exhibited consistent roughness values in all three measurements (Figure 9c), resulting in a Ra = 4.89 ± 0.05 nm and Rq = 6.39 ± 0.36 nm, whereas for the microspheres, the mean roughness values were greater as follows: Ra = 295.33 ± 7.93 nm and Rq = 356.33 ± 9.28 nm. The significant difference between the roughness values indicates a more controlled and precise mode of action for the nanocapsules, potentially making them more efficient for specific tasks.

An average interparticle spacing of approximately 150–200 nm was observed in the analyzed AFM images, indicating that the nanoscale surface features were generally well-separated within the dried deposit, as confirmed by the three-dimensional profile shown in Figure 9e. The apparent lateral diameter distribution of these surface features (Figure 9f) followed a narrow Gaussian distribution, with a mean value of 100 ± 10 nm, suggesting good uniformity of the nanoscale structures observed in the dried film.

Antioxidant activity results indicate that free quercetin retained the highest activity, while encapsulated forms showed reduced activity. This decrease is likely due to partial shielding of the active compound within the polymer matrix, limiting its interaction with ABTS radicals.

Q1 showed moderate activity (IC_50_ = 5.1 µg/mL), approximately ten times weaker than the quercetin control. However, it was approximatively four times more active than Q2 (IC_50_ = 17.6 µg/mL). This difference may be attributed to variations in nanocapsule composition, encapsulation efficiency, or the release behavior of quercetin. The reduced antioxidant activity observed in nanocapsule formulations compared with free quercetin may be explained by partial shielding of the active compound within the polymer matrix, which limits its direct interaction with ABTS•^+^ radicals. Additionally, comparisons were performed based on the total sample mass (µg/mL), without considering the actual quercetin content, which may lead to an underestimation of the intrinsic antioxidant capacity of encapsulated quercetin.

Overall, nanocapsules provide a more controlled and potentially efficient delivery system, while microspheres are more suitable for sustained release applications.

The changes observed in the crystalline structure after formulation indicate the influence of the encapsulation process on the physical organization of quercetin within the carrier system. The transition from a crystalline arrangement to a predominantly amorphous state is generally associated with improved molecular dispersion of the bioactive compound and stronger interactions with the surrounding polymeric components [35,40]. Such structural modifications can be beneficial for quercetin, considering its limited aqueous solubility, as the reduction in crystallinity may favor its dissolution behavior and facilitate its availability in subsequent applications [30,40]. The differences observed among the formulations suggest that the composition and preparation conditions played an important role in controlling the molecular organization of quercetin within the matrix. In particular, the fully amorphous character of Q3 may indicate a more pronounced disruption of the original crystalline arrangement, which could be related to the distinct formulation approach used for this sample. Overall, the XRD findings support the formation of a modified quercetin delivery system in which the bioactive compound is effectively dispersed within the carrier matrix [30,35].

The encapsulation performance of the quercetin-loaded microspheres/nanocapsules was evaluated based on the encapsulation efficiency (EE) and loading capacity (LC) determined by HPLC analysis. The obtained results showed EE values of 28.18%, 51.79%, and 23.95% for the Q1, Q2, and Q3 formulations, respectively, while the corresponding quercetin loading capacities were 4.94%, 5.78%, and 2.50%. Among the investigated formulations, Q2 exhibited the highest encapsulation efficiency and loading capacity, indicating a more efficient incorporation of quercetin into the polymeric matrix. The increased quercetin retention observed for Q2 may be attributed to the specific formulation parameters and preparation conditions, which could favor stronger interactions between quercetin and the encapsulating materials, as well as improved entrapment within the polymer network [30,42].

In comparison, Q1 showed a lower EE despite containing the same initial amount of quercetin, suggesting that the encapsulation process was influenced not only by the amount of bioactive compound added, but also by the physicochemical characteristics of the formulation [30,42]. Q3 presented lower EE and LC values, which can be explained by the different preparation procedure and the lower initial quercetin concentration used in this formulation [40]. The differences in EE and LC among the formulations highlight the importance of optimizing the quercetin-to-polymer ratio and processing parameters to achieve efficient encapsulation and maximize the amount of active compound delivered by the microspheres/nanocapsules [35,42].

Encapsulation efficiency and antioxidant activity did not correlate directly among the investigated formulations, indicating that these parameters describe different aspects of quercetin performance. Q2 exhibited the highest encapsulation efficiency (51.79%) and loading capacity (5.78%), demonstrating superior quercetin retention within the polymeric matrix. However, despite its higher encapsulation performance, Q2 showed a lower antioxidant activity (IC_50_ = 17.6 µg/mL) than Q1 (IC_50_ = 5.1 µg/mL) [30,35,42].

This difference can be explained by the fact that encapsulation efficiency reflects the amount of quercetin retained within the carrier, whereas the ABTS assay measures only the fraction immediately available to interact with free radicals. Therefore, the higher encapsulation efficiency of Q2 likely resulted in greater shielding of quercetin within the polymer matrix, reducing its apparent antioxidant activity while potentially improving its protection and controlled-release properties. These findings are consistent with the AFM and XRD analyses, which indicated a well-organized nanostructure and the improved molecular dispersion of quercetin after encapsulation. Overall, Q2 appears more suitable for controlled delivery applications, whereas Q1 provides greater immediate antioxidant activity due to the higher accessibility of quercetin during the assay.

## 4. Materials and Methods

All chemicals used in the formulation of microspheres, including poly(lactic acid) (PLA), poly(vinyl alcohol) (PVA), and dichloromethane (DCM), were purchased from Sigma-Aldrich (Steinheim, Germany). For the synthesis of nanocapsules, poloxamer 407, ethyl acetate, polycaprolactone diol, and 0.1 N sodium hydroxide were also obtained from Sigma-Aldrich (Steinheim, Germany). Miglyol^®^ 810 N was supplied by IOI Oleochemical (Hamburg, Germany). Quercetin hydrate > 95% purity powder was purchased from Sigma-Aldrich (Steinheim, Germany), and whey from bovine milk was lyophilized at ICCRR-laboratory, Romania.

### 4.1. Preparation of Whey–Quercetin Nanocapsules (Q1, Q2)

The experimental formulations, including the whey–quercetin nanocapsules (Q1 and Q2) and PLA microspheres (Q3), together with their preparation methods, are presented in Table 4. Whey–quercetin nanocapsules (Q1 and Q2) were prepared using the interfacial polymerization technique. The aqueous phase was prepared by dissolving 2.5 g of poloxamer in 100 mL of distilled water. This solution was then added dropwise to the ethyl acetate solution using a dropping funnel.

The organic phase consisted of 30 mL of ethyl acetate containing whey (2 g for Q1 and 5 g for Q2), 1 g of quercetin, 0.2 g of polycaprolactone (PCL), 1.2 mL of Miglyol, and 0.6 mL of distilled water. This phase was added dropwise to the aqueous phase using a micropipette under continuous magnetic stirring at 800 rpm for 15 min. The pH of the system was adjusted from 5.0 to neutral using a 0.1 M NaOH solution. Subsequently, ethyl acetate was removed by rotary evaporation. The resulting nanocapsule suspension was then lyophilized and subjected to further characterization. All samples were prepared and analyzed in three independent replicates.

### 4.2. Preparation of PLA/Whey/Quercetin Microspheres (Q3)

PLA microspheres encapsulating quercetin and whey were prepared using the sol-id-in-oil-in-water (S/O/W) emulsification technique. The organic phase was prepared by dissolving 1 g of PLA in 10 mL of dichloromethane, while the aqueous phase consisted of 4 g of poly(vinyl alcohol) (PVA) dissolved in 100 mL of distilled water.

The solid phase, containing 2 g of whey and 0.06 g of quercetin, was dispersed in the organic phase to form an S/O suspension. This suspension was homogenized at 5000 rpm for 30 s and subsequently added dropwise to a 4% (*w*/*v*) PVA aqueous solution. The resulting S/O/W emulsion was further homogenized at 7000 rpm for 10 min using a magnetic stirrer.

The organic solvent was then removed by extraction through immersion of the emulsion in 2 L of deionized water for 30 min. Finally, the microspheres were collected by filtration and dried at room temperature for further characterization. All samples were prepared and analyzed in triplicate.

### 4.3. Polarized Light Microscopy (PLM)

Optical microscopy was conducted with an Laboval 2 microscope (Zeiss Company, Oberochen, Germany) in cross polarized light and transmitted light. Digital images were acquired with a system Samsung 10 MPx (Samsung, Suwon, Republic of Korea) attached to the optical microscope.

### 4.4. SEM Microscopy

The nanocapsules were characterized using a scanning electron microscope (Inspect™ S, FEI Company, Hillsboro, OR, USA). SEM observations were carried out under low-vacuum conditions at an accelerating voltage of 20 kV. The samples were examined at magnifications of 500, 1000, 2000, and 5000×.

Particle diameter distribution was quantified on SEM and AFM images using Image J software version 1.53 k (National Institutes of Health, Bethesda, MD, USA). The particle size quantification thresholds were defined according to each analyzed image, considering the starting value (e.g., usually 0) and the end value, along with the particle size step.

### 4.5. Atomic Force Microscopy (AFM)

Topographical images were acquired in AC (tapping) mode using an atomic force microscope (AFM; JEOL 4210, JEOL Ltd., Tokyo, Japan). In this mode, the cantilever tip intermittently contacts the surface, which is the most commonly employed operating mode in AFM. Surface scanning was performed using NSC15 cantilevers (MikroMasch, Tallinn, Estonia) with a nominal resonant frequency of 325 kHz and a spring constant of 40 N/m. The acquired images were processed using JEOL WIN SPM software (JEOL Ltd., Tokyo, Japan).

### 4.6. Statistical Analysis

The statistical analysis of the roughness values was conducted using Origin Lab software version 2018b (Microcal Company, Northampton, MA, USA) using the ANOVA test with Tukey post hoc analysis at a significance level of a = 0.05; *p* values lower than the significance value reveal relevant statistical differences while *p* values higher than the significance level have statistical similarities forming a statistical relevant group.

### 4.7. Fourier Transform Infrared (FTIR) Spectroscopy

The chemical bonding in the synthesized microspheres and nanocapsules was examined by Fourier transform infrared (FTIR) spectroscopy using an FTIR-610 spectrometer (Jasco Corporation, Tokyo, Japan) equipped with an attenuated total reflectance (ATR) accessory incorporating a horizontal ZnSe crystal (Jasco PRO400S). Samples were directly applied onto the ZnSe crystal, and spectra were collected over the 4000–500 cm^−1^ range with a resolution of 4 cm^−1^. Each spectrum represents the average of 100 scans, and all measurements were carried out at room temperature.

### 4.8. Oxidation of Compounds by Chemically Generated ABTS•^+^ Radical

ABTS and potassium persulfate were dissolved separately in deionized water at concentrations of 7 mM and 20 mM, respectively. These solutions were mixed to obtain 5 mM ABTS and 2.5 mM K_2_S_2_O_8_, and the mixture was incubated in the dark at room temperature for 12–16 h to generate the ABTS•^+^ radical cation [45]. Then, different concentrations of the tested compounds were mixed with the ABTS•^+^ solution in 1 mL of 50 mM sodium phosphate buffer, pH 7.0. ABTS•^+^ was used at a concentration that gave an initial absorbance of 0.65 (approximately 20 µM). The compounds subjected to analysis were prepared as stock solutions at a concentration of 1 mg/mL, using appropriate solvents according to their chemical properties. Specifically, standard quercetin and sample 1 (Q) were dissolved in ethanol, while sample 2 (W) and nanocapsule samples 3 (Q1) and 4 (Q2) were dissolved in distilled water. Sample 5 (Q3), characterized by lower polarity, was dissolved in acetonitrile. After dissolution, the stock solutions were diluted with 50 mM sodium phosphate buffer to a concentration of 0.1 mg/mL.

A calibration curve for quercetin was constructed over the concentration of 0.2 and 0.8 µg/mL. Absorbance spectra were recorded for 30 min over the wavelength range of 230–900 nm using a Lambda 25 spectrophotometer (PerkinElmer, Singapore). Quantitative analysis was performed using the absorbance measured at 415 nm.

### 4.9. X-Ray Diffraction (XRD)

X-ray diffraction measurements were performed with a D8 Advance X-ray diffractometer (Bruker Company, Karlsruhe, Germany) equipped with a monochromator in the incident beam in order to obtain only Cu Kα1 radiation. The X-ray tube was powered at 40 kV and 40 mA, and the diffracted beam was recorded with a LynxEye position detector (Bruker AXS GmbH, Karlsruhe, Germany). The measurements were performed in the 3–49° range in steps of 0.02°. Diffraction patterns are shown in Figure 12.

### 4.10. HPLC Measurements

The analyses were performed on a Jasco (Japan) HPLC chromatograph equipped with a HPLC pump (Model PU-980), a ternary gradient unit (Model LG-980-02), a thermostat column (Model CO-2060 Plus), a UV–Vis detector (Model UV-975), and an injection valve equipped with a 20 μL sample loop (Rheodyne, Rohnert Park, CA, USA). Samples were manually injected with a Hamilton Rheodyne syringe (50 mL) (Hamilton Company, Reno, NV, USA). The HPLC system was controlled, and the experimental data were analyzed with the software ChromPass (version 1.8). All determinations were performed in triplicate, and the data are presented as the mean ± standard deviation (SD).

### 4.11. DSC Measurements

Differential scanning calorimetry (DSC) measurements were conducted using a DSC 630e calorimeter (Mettler-Toledo, Singapore), capable of operating at temperatures up to 750 °C. The analyses were performed under a continuous nitrogen atmosphere using 40 μL aluminum crucibles. Samples were heated from 25 to 250 °C at a constant heating rate of 10 °C/min, followed by an isothermal stage of 0.5 min at the maximum temperature. Throughout the experiments, the nitrogen purge was maintained at a flow rate of 80 mL/min. The thermal transition parameters, including the glass transition temperature (Tg), crystallization temperature (Tc), and melting temperature (Tm), were determined from the resulting DSC thermograms.

## 5. Conclusions

The present study demonstrates the successful development of whey–quercetin microspheres and nanocapsules using the solid-in-oil-in-water (S/O/W) emulsification method and interfacial polymerization. Morphological and structural characterization by OMM, SEM, AFM, and FTIR confirmed the successful encapsulation of the bioactive compounds within the prepared systems. Although both formulations achieved the successful incorporation of whey and quercetin, only the nanocapsules exhibited significantly higher antioxidant activity, which was substantially higher than that of the microspheres under the experimental condition. These findings indicate that whey–quercetin nanocapsules represent a promising platform for the stabilization and controlled delivery of antioxidant compounds, highlighting their potential applicability in pharmaceutical, nutraceutical, or functional food formulations.

The results demonstrate a trade-off between encapsulation performance and immediate antioxidant activity. Nanocapsules containing 5 g of whey appear to be the most suitable formulation for applications requiring efficient quercetin protection and sustained release, whereas nanocapsules containing 2 g of whey exhibit greater short-term antioxidant activity due to the higher availability of quercetin during the assay.

Therefore, the optimal formulation should be selected according to the intended application, balancing antioxidant efficacy with controlled release and the stability of the bioactive compound.

## Figures and Tables

**Table 1:** IC_50_ values for compounds with antioxidant activity.

Sample	IC_50_ (µg/mL)
Quercetin	0.6
Whey	No activity
Q1	5.1
Q2	17.6
Q3	No activity

(IC_50_ = concentration of compound at which half of the radicals are inhibited at t = 30 min).

**Table 2:** HPLC analysis of quercetin content in the whey–quercetin nanocapsules and PLA microspheres.

Sample	Quercetin (mg/g)
Q1	49.39 ± 1.08
Q2	57.84 ± 1.23
Q3	24.78 ± 0.69

**Table 3:** Encapsulation efficiency (EE) and loading capacity (LC) of the quercetin-loaded nanocapsules and microspheres.

Sample	Loading Capacity (%)	Encapsulation Efficiency (%)
Q1	4.94 ± 0.53	28.18 ± 0.87
Q2	5.78 ± 0.71	51.79 ± 1.03
Q3	2.48 ± 0.32	24.40 ± 0.59

**Table 4:** The experimental samples of the whey–quercetin nanocapsules (Q1, Q2) and PLA microspheres (Q3).

Sample	Formulation Type	Preparation Method
Q1	Nanocapsules	Interfacial polymerization
Q2	Nanocapsules	Interfacial polymerization
Q3	Microspheres	Solid-in-oil-in-water (S/O/W)

## Data Availability

The original contributions presented in the study are included in the article; further inquiries can be directed to the corresponding author.

## Abbreviations

The following abbreviations are used in this manuscript:

PLA	Polylactic acid
PVA	Polyvinyl alcohol
DCM	Dichloromethane
PLM	Polarized light microscopy
SEM	Scanning electron microscopy
AFM	Atomic force microscopy
FTIR	Fourier transform infrared spectroscopy
HPLC	High-performance liquid chromatography
XRD	X-ray diffraction
DSC	Differential scanning calorimetry
